# Anisometropic Patient and Current Bioelectrical Activity in the Masticatory and Cervical Muscles

**DOI:** 10.3390/reports7030054

**Published:** 2024-07-10

**Authors:** Grzegorz Zieliński, Anna Woźniak, Michał Ginszt, Jacek Szkutnik, Nicola Marchili, Marcin G. Prost, Piotr Gawda, Robert Rejdak

**Affiliations:** 1 Department of Sports Medicine, Medical University of Lublin, 20-093 Lublin, Poland; 2 Department of General and Pediatric Ophthalmology, Medical University of Lublin, 20-093 Lublin, Poland; 3 Department of Rehabilitation and Physiotherapy, Medical University of Lublin, 20-093 Lublin, Poland; 4 Independent Unit of Functional Masticatory Disorders, Medical University of Lublin, 20-093 Lublin, Poland; 5 Private Practice in Rieti, 02100 Rieti, Italy; 6 Stolmed Oogklinieken B.V., Voorsteven 48-50, 4871 DX Etten-Leur, The Netherlands

**Keywords:** myopia, hyperopia, astigmatism, anisometropia, amblyopia, sEMG, TMJ, muscles, masticatory muscles, cervical muscles

## Abstract

(1) Background: This study aims to analyze the bioelectrical activity of the masticatory and cervical muscles in a subject with anisometropia. (2) Methods: A female patient aged 23 years with a best-corrected visual acuity of 1.0 in the right eye and 0.1 in the left eye, a refractive error of −2.25 Dsph in the right eye and +4.25 Dsph in the left eye, and astigmatism of −1.75 Dcyl axis 24° was examined. A comprehensive ophthalmological examination and the study of the bioelectrical activity of the muscles were carried out. During the ophthalmological examination, best-corrected visual acuity was determined, refractive error (spherical equivalent) was assessed, and additionally, retinal thickness, choroidal thickness, axial length, and intraocular pressure were measured. (3) Results: It was demonstrated that higher tension in the resting mandibular position and pain-free maximum unassisted opening were observed on the right side (myopia). Conversely, higher tension during maximum voluntary clenching in the intercuspal position and maximum voluntary clenching on dental cotton rolls in the intercuspal position was observed on the left side (hyperopia and astigmatism). (4) Conclusions: In the case study, muscle asymmetry was demonstrated, which is likely associated with anisometropia. This phenomenon requires further investigation.

## 1. Introduction

Anisometropia is a condition characterized by a significant difference in the refractive error between the two eyeballs [1,2]. It can lead to variations in visual acuity between the eyes, causing difficulties with depth perception, binocular vision, visual acuity, and amblyopia. Anisometropia can occur with varying degrees of severity and can be corrected with eyeglasses, contact lenses, or, in some cases, refractive surgery [1,2]. Based on research carried out across various geographical areas, the prevalence of this varies between 3.79% and 21.8% [1].

Typically, a difference in the power of one diopter is the threshold for diagnosing this condition [3]. Patients can have up to three diopters of anisometropia before the condition becomes clinically significant. Reasons for this can include headaches, double vision, or photophobia. In some types of anisometropia, the visual cortex of the brain is unable to process images from both eyes simultaneously. This often occurs within the first 10 years of life in individuals with this condition, as the visual cortex develops, which can lead to amblyopia [4]. Antimetropia (occurring in about 0.1% of student populations) is a rare subtype of anisometropia, where one eye is myopia, and the other is hyperopia [5].

In the two literature reviews, functional connections between the visual and muscular systems were demonstrated [6,7], apart from functional connections manifested as changes in the bioelectrical activity of muscles in response to visual stimuli (such as opening versus closing eyes in individuals with nearsightedness) [8]. Additionally, correlations have been found between the extraocular muscles and the muscles of the masticatory system [9].

The phenomenon of changes in the bioelectrical activity of the muscles of the masticatory organ in people with myopia seems to have been fairly well studied [6,7,8,9]. However, there have been no studies of changes in muscle bioelectrical activity in people with anisometropia. This study aimed to analyze the bioelectrical activity of the masticatory and cervical muscles in subjects with anisometropia.

## 2. Detailed Case Description

Firstly, surface electromyography (sEMG) examination was conducted, analyzing the bioelectrical activity of the masticatory muscles (temporalis muscle, masseter muscle, and digastric muscle) as well as the cervical spine muscles (sternocleidomastoid muscle). An examination was carried out according to the standard procedure of 10 s of resting mandibular position activity and testing the maximum voluntary clenching in the intercuspal position, maximum voluntary clenching on dental cotton rolls, and pain-free maximum unassisted opening (3 s of activity, 2 s of rest, repeated three times) [10]. Recordings were analyzed with the eyes open (uncorrected) and with the eyes closed [8].

An 8-channel electromyograph BioEMG III™ was used for the examination. Microvolt signals were amplified, virtually without noise, to 5000 times their original levels. The amplifier had an impedance greater than 100 MegOhms. It could handle input signals from 0 to 2000 microvolts (peak to peak). The amplifier had low noise, producing less than 0.3 microvolts on average. The DC offset range of the amplifier was from −0.7 volt to +0.7 volt. In common-mode rejection, with a ratio greater than 130 dB at 60 Hertz and greater than 120 dB from 100 to 600 Hertz. It handled common-mode voltages ranging from −6.5 to +6.5 volts DC. The amplifier had a bandwidth of 30 to 1000 Hertz when operating at a 2000 Hertz sample rate. Sensitivity was less than 0.3 microvolts (peak to peak). The resolution of the A/D converter was 0.5 microvolts. It provided silent period measurements to the nearest millisecond. It could withstand a maximum peak voltage of 3500 volts when applied [11,12,13]. 

Software Noise Reduction is an essential feature in various electronic devices and equipment, including electromyographs like the BioEMG III. In this context, the BioEMG III electromyograph incorporates software Noise Reduction (BioPAK Measurement System, BioResearch Associates, Inc., Milwaukee, WI, USA), with a nominal noise reduction of 40 dB. To put this into perspective, a reduction of 40 dB on a logarithmic scale translates to an impressive 99% reduction in noise amplitude when viewed on a linear scale. This means that the device effectively minimizes unwanted noise, ensuring that the recorded signals are exceptionally clean and accurate for precise analysis and diagnosis [11,12,13].

For bioelectrical activity analyses, the asymmetry index was additionally used. The asymmetry index (AsI) was calculated based on the Root Mean Square (RMS) to evaluate the imbalance in muscle activity between the right and left sides of the masticatory organ. AsI values can range from +100% to −100%, where +100% indicates exclusive muscle activity on the right side and −100% indicates exclusive activity on the left side. A value of 0% signifies equal muscle activity on both sides. This index, introduced by Naeije et al. [14], is calculated using the following formula:

Asymmetry index (AsI) = (RMS_right_ − RMS_left_)/(RMS_right_ + RMS_left_) × 100

Subsequently, an ophthalmic examination was conducted, consisting of the Snellen chart to examine the best-corrected visual acuity [15], refraction testing (Topcon KR-800 autokeratorefractometer test, Topcon Co., Tokyo, Japan) [16], determination of ocular length (IOL Master 500 equipment, Carl Zeiss Meditec, Jena, Germany) [17], determination of choroidal and retinal thickness (optical coherence tomography, Optovue AngioVue, Fremont, CA, USA) [18], and intraocular pressure (Tono-Pen XL, Medtronic Solan, FL, USA) [19].

An examination for temporomandibular disorders (TMDs) assessment according to the Polish version of the DC/TMD examination was also performed [20].

### Case Study

A female patient aged 23 years was examined. Her best-corrected visual acuity (BCVA) was 20/20, with −2.25 D in the right eye and 0.1 with +4.25 D/−1.75 Dcyl ax 24° in the left eye. The patient came from a rural area and was diagnosed with refractive error at the age of 5 years. The anterior and posterior segments of both eyes and intraocular pressure were normal. No strabismus was revealed. Her parents also had refractive errors: her mother has hyperopia, and her father has myopia. She was not diagnosed with TMD, and her mandibular range of motion is shown in Table 1. The patient was not diagnosed with any occlusal abnormalities or oral diseases.

During the sEMG examination, it was found that there was higher resting tension on the myopic eye side compared to the hyperopic eye side (the open eyes test). A decrease in bioelectrical activity was observed during the test conducted with closed eyes regardless of the side. However, higher tension persisted on the myopic eye side.

Changes occurred during maximum voluntary clenching in the intercuspal position and maximum voluntary clenching on dental cotton rolls in the intercuspal position—higher bioelectrical tension was consistently observed on the hyperopic eye side. Closing the eyes also resulted in a decrease in the bioelectrical activity of the muscles examined.

During pain-free maximum unassisted opening, higher bioelectrical tension was observed on the right side (myopic). Closing the eyes also resulted in a decrease in the bioelectrical activity of the examined muscles. All sEMG recordings showed masseter muscle hyperactivity (Table 2).

The analysis of AsI showed a decrease in muscle asymmetry in the majority of patients during this study with their closed eyes (Table 3). A change in the index towards greater muscle symmetry was demonstrated in the TA and MM in the resting mandibular position; MM and DA during clenching; SCM and DA during clenching on dental cotton rolls; and TA and MM during pain-free maximum unassisted opening. An increase in asymmetry occurred only in the neck muscles, specifically in SCM and DA in the resting mandibular position and SCM during pain-free maximum unassisted opening.

## 3. Discussion

This study aimed to analyze the bioelectrical activity of the masticatory and cervical muscles in subjects with anisometropia. It was demonstrated that higher tension in the resting mandibular position and the pain-free maximum unassisted opening were observed on the right side (myopia). Conversely, higher tension during maximum voluntary clenching in the intercuspal position and maximum voluntary clenching on dental cotton rolls was observed on the left side (hyperopia and astigmatism) (Table 2). The results were confirmed by the asymmetry index results (Table 3).

To the best of our knowledge, individuals with anisometropia have not been comprehensively examined regarding changes in the bioelectrical activity of their muscles so far, despite this being performed for other refractive errors [6,7,8]. The patient had experienced a visual deterioration since the age of five. The left eye, with a greater refractive error, had been affected by amblyopia. Amblyopia is a condition where, even when correcting the refractive error properly, the vision in the affected eye may still not be fully correctable to 20/20. This is caused by the lack of normal procession of images from both eyes simultaneously by the visual cortex. As a result of this, the brain suppresses the vision of the worst eye. This occurs in childhood, often during the first decade of life, while the visual cortex is developing.

Hypothetically, the left-side bioelectrical dominance during clenching and tightening on cotton rolls may be explained by the fact that resting tension is combined with contractile activity. During rest (for example, low tension), if a muscle fiber is not at its optimal length, it might not generate maximal tension during contraction. The optimal tension is reached when muscle fibers have the correct overlap of actin and myosin, allowing for the formation of the maximum number of cross bridges. If a muscle is overly stretched or shortened beyond this optimal length, the number of effective cross-bridges that can form during contraction decreases, leading to reduced tension generation [21,22,23].

The effect of particularly suspended resting tension in the right eye can be explained by the visual-motor stabilization effect. The visual-motor stabilization effect refers to the phenomenon where the visual system stabilizes or adjusts perceptual input in response to motor actions or movements, particularly during tasks requiring precise motor control. In other words, it describes the tendency of the visual system to stabilize visual perception to maintain a stable visual image while engaging in motor activities [24,25]. The observed decrease in bioelectrical activity during eye closure aligns with current research findings [6].

The phenomenon itself and the quite noticeable muscle asymmetry require further investigation in a larger study group. This is especially significant in terms of the prevention and treatment of diseases that may potentially be associated with muscle asymmetry (tension-type headaches [26], TMDs [27,28], etc.).

## 4. Conclusions

In the case study, muscle asymmetry was demonstrated, which is likely associated with anisometropia. This phenomenon requires further investigation.

## Figures and Tables

**Table 1:** Presentation of ophthalmic and dental findings.

	Right	Left
Age	23	
Best-corrected visual acuity	20/20	20/200
Refractive error (dsph)	−2.25	+4.25
Astigmatism	0	−1.75
Intraocular pressure (mmHg)	20	20
Retinal thickness (μm)	228	257
Choroidal thickness (μm)	314	395
Axial length (mm)	24.14	21.67
Mandibular range of motion (mm)		
Pain-free opening	49	
Maximum unassisted opening	49	
Maximum assisted opening	51	
Mandibular movement to the right	9	
Mandibular movement to the left	8	
Protrusion	8	

**Table 2:** Presentation of results of bioelectrical activity of the examined muscles.

		Right		Left	
		Eye Open	Eye Closed	Eye Open	Eye Closed
Resting mandibular position (μV)	TA	3.23	2.1	1.99	1.79
	MM	5.69	3.81	4.68	3.64
	SCM	1.69	1.44	1.49	1.22
	DA	1.85	1.740	1.56	1.35
Maximum voluntary clenching in the intercuspal position (μV)	TA	139.90	130.60	189.5	176.80
	MM	313.50	277.40	353.00	299.50
	SCM	18.40	15.40	15.90	13.40
	DA	33.40	31.50	44.40	38.00
Maximum voluntary clenching on dental cotton rolls (μV)	TA	155.80	143.70	217.20	200.50
	MM	298.50	254.90	311.40	269.20
	SCM	18.40	13.10	13.30	13.00
	DA	31.20	28.90	37.90	33.00
Pain-free maximum unassisted opening (μV)	TA	7.40	5.20	3.80	4.20
	MM	7.10	6.70	5.20	5.50
	SCM	8.20	13.00	6.80	9.00
	DA	74.40	55.00	72.30	57.00

TA—the temporalis anterior; MM—the superficial part of the masseter muscle; SCM—the middle part of the sternocleidomastoid muscle belly; and DA—the anterior belly of the digastric muscle.

**Table 3:** Results of the asymmetry index.

		Eye Open	Eye Closed	
Resting mandibular position (μV)	TA	0.24	0.08	−
	MM	0.10	0.02	−
	SCM	0.06	0.08	+
	DA	0.09	0.13	+
Maximum voluntary clenching in the intercuspal position (μV)	TA	−0.15	−0.15	=
	MM	−0.06	−0.04	−
	SCM	0.07	0.07	=
	DA	−0.14	−0.09	−
Maximum voluntary clenching on dental cotton rolls (μV)	TA	−0.16	−0.17	=
	MM	−0.02	−0.03	=
	SCM	0.16	0.00	−
	DA	−0.10	−0.07	−
Pain-free maximum unassisted opening (μV)	TA	0.32	0.11	−
	MM	0.15	0.10	−
	SCM	0.09	0.18	+
	DA	0.01	−0.02	−

“=”—no change in the asymmetry index (torelance—0.01), “−”—decrease in asymmetry, and “+”—increase in asymmetry”.

## Data Availability

The datasets generated during and analyzed during the current study are available from the corresponding author on reasonable request.
